# WHO guidelines on fluid resuscitation in children: missing the FEAST data

**DOI:** 10.1136/bmj.f7003

**Published:** 2014-01-14

**Authors:** Sarah Kiguli, Samuel O Akech, George Mtove, Robert O Opoka, Charles Engoru, Peter Olupot-Olupot, Richard Nyeko, Jennifer Evans, Jane Crawley, Natalie Prevatt, Hugh Reyburn, Michael Levin, Elizabeth C George, Annabelle South, Abdel G Babiker, Diana M Gibb, Kathryn Maitland

**Affiliations:** 1 Department of Paediatrics, Mulago Hospital, Makerere University, Kampala, Uganda; 2 Kilifi Clinical Trials Facility, KEMRI-Wellcome Trust Research Programme, Kilifi, Kenya; 3 Department of Paediatrics, Joint Malaria Programme, Teule Hospital, Muheza, Tanzania; 4 Department of Paediatrics, Soroti Regional Referral Hospital, Soroti, Uganda; 5 Department of Paediatrics, Mbale Regional Referral Hospital Pallisa Road, Mbale, Uganda; 6 Department of Paediatrics, St Mary’s Hospital, Lacor, Uganda; 7 Department of Paediatrics University Hospital of Wales Heath Park, Cardiff, UK; 8 Nuffield Department of Medicine, University of Oxford, Oxford, UK; 9 Wellcome Trust Centre for Clinical Tropical Medicine, Department of Paediatrics, Faculty of Medicine, St Mary’s Campus, London W2 1PG, UK; 10 Joint Malaria Programme, Moshi, Tanzania; 11 Medical Research Council Clinical Trials Unit, UCL, London, UK

The World Health Organization recommendations on management of common childhood illnesses affect the lives of millions of children admitted to hospital worldwide. Its latest guidelines,^1^ released in May 2013, continue to recommend rapid fluid resuscitation for septic shock, even though the only large controlled trial of this treatment (Fluid Expansion as a Supportive Treatment (FEAST) found that it increased the risk of death in African children.^2^ A subsequent systematic review of bolus resuscitation in children with shock resulting from severe infection also did not support its use.^3^ Failure to take this evidence into account is not consistent with WHO’s commitment to systematically and transparently assess evidence using the GRADE (Grading of Recommendations Assessment, Development and Evaluation) process when producing guidelines and could endanger the lives of children.

## Evidence on fluid resuscitation

Rapid fluid resuscitation was recommended as a lifesaving treatment for shock on the basis of a GRADE systematic review that found weak evidence of benefit (largely expert opinion based on two paediatric case series at a single tertiary centre).^4^ It has become a key component of surviving sepsis campaigns in children and adults^4^ ^5^ and is widely practised in well resourced settings. Fluid resuscitation is also being increasingly promoted in resource poor settings^6^ ^7^ as part of the WHO endorsed emergency triage assessment and treatment training.^8^ This is despite systematic reviews^9^ and commentaries highlighting concerns that these recommendations are not based on research evidence.^10^

FEAST was published in 2011. It is the only randomised controlled trial comparing bolus fluid resuscitation with no bolus. The study was conducted in six African hospitals without intensive care facilities in Kenya, Tanzania, and Uganda and enrolled 3141 children with fever and shock (one or more features of impaired perfusion with impaired consciousness or respiratory distress, or both). The study included a prespecified analysis of subgroups of children with malaria and anaemia, as these conditions are relevant to resource poor settings. Children with gastroenteritis, severe malnutrition, burns, or surgical conditions were excluded.

Children were randomly assigned to receive rapid resuscitation with albumin or normal saline boluses (20-40 ml/kg over 1-2 hours) or no bolus (control group). All children received standard treatments according to their illness, including standard of care maintenance fluids (mainly 5% dextrose/saline at 2.5-4 ml/kg/h) until able to drink, antibiotics, antimalarials, oxygen, and transfusion.

The trial was stopped early by the data monitoring committee because rapid resuscitation resulted in a 45% relative (95% confidence interval 13% to 86%) increase in 48 hour mortality compared with controls. The absolute excess in mortality was 3.3% (1.2% to 5.3%). This increase in mortality was seen in every subgroup, across the age spectrum (3 months-12 years), and at each of the six centres from three countries in the trial,^2^ irrespective of the pathogen (malaria, bacterial sepsis, or anaemia). Further planned analysis showed that although children given a bolus had a superior shock resolution than those in the control group, they were more likely to die as a result of cardiovascular collapse.^11^

A systematic review published in 2012 assessed the evidence for bolus fluid resuscitation further and included 13 studies (four in general shock, four in malaria, four in dengue fever, and one in severe malnutrition).^3^ The only study to include a control arm (no fluid bolus) was FEAST, which drove the results. Overall, and in subgroups of children with sepsis or malaria, those who received no fluid bolus had significantly lower mortality at 48 hours (76/1044) compared with those who received saline or colloid boluses (221/2097, relative risk 0.69, 95% confidence interval 0.54 to 0.89 for sepsis and 0.64, 0.46 to 0.91 for malaria**).**

## Problems interpreting FEAST

A serious question raised during the debate about FEAST was whether the broad criteria used to define shock affected the applicability of the results since various international guidelines use a narrower definition of shock, which in turn may influence how children are managed.^12^–^14^ FEAST defined shock as children with fever and one or more features of impaired perfusion plus impaired consciousness or respiratory distress, or both. Half of the children had two or more features. But within this broad definition we were able to look at subgroups that meet the narrower criteria used in US and WHO guidelines.^5^–^15^ We applied all published definitions of paediatric shock to the FEAST trial data (table⇓)^12^ and found that for every definition, bolus resuscitation resulted in a worse outcome compared with control.

The criteria for shock in the WHO guidelines represent the sickest children, requiring the presence a capillary refilling time of more than 3 seconds, cold peripheries, a weak pulse, and a fast pulse. This definition applied to only 65 (2%) of the 3141 children in FEAST. They were a very high risk group, accounting for about 10% of all deaths in the trial; 24/50 (48%) of children who received boluses died within 48 hours compared with 3/15 (20%) of control children meeting WHO criteria^12^—an absolute increase in risk of 28% and relative risk of 240% (P=0.07, two sided Fisher’s exact test). Although the FEAST trial was not powered to detect differences between arms for children in the WHO defined shock, a basic principle of clinical trials is that subgroup results should be interpreted within the context of the overall trial results, which provide a more reliable assessment of the effect of the intervention than an analysis restricted to patients in the subgroup.^16^ ^17^ The result in the subgroup is consistent with the overall result.

Concern has also been expressed about the consequences of not giving bolus fluids to children with moderate hypotension and severe dehydration. Again, FEAST was not powered to detect differences in these subgroups, but the results are consistent with harm from use of bolus resuscitation.^12^

In children with hypotension (defined in FEAST as systolic blood pressure 50-75, 60-75, and 70-85 mm Hg in children aged <12 months, 1-5 years, and >5 years respectively, in line with clinical use) there was a trend towards increased mortality in the bolus arms (absolute difference 9.4%, 95% confidence interval −2.6% to 21.4%). Severe hypotension is very uncommon in children, as shown by the very small number of children (n=29) with this condition who were enrolled in the FEAST trial; all of these children were randomised to receive either colloid of saline boluses.^2^ Of interest, only eight of the 29 children fulfilled the WHO definition of shock and all eight died.

Overall, severe dehydration without diarrhoea was present in 236 children (7.5%) in FEAST, and we found no evidence that boluses were of benefit; there were 38/173 (22%) deaths in the bolus arm versus 8/58 (13.8%) in the control (relative risk 1.59, 95% confidence interval 0.79 to 3.21).^11^

## Change led by FEAST

The FEAST trial was praised for demonstrating how rigorous clinical research can be done in resource poor settings. Subsequently, the findings have been widely debated, as they challenged the primacy of bolus resuscitation as a lifesaving intervention for paediatric shock in resource limited settings and raised questions about their use elsewhere. Following publication of a systematic review of the evidence,^3^ Médicins Sans Frontières revised its paediatric shock guideline in March 2012.

A meeting hosted by the Kenyan Paediatric Association in October 2012 raised concern about WHO’s lack of response to the FEAST results. Participants, including representatives from 10 countries in sub-Saharan Africa, sent a letter to WHO in March 2013, stating that they had reviewed the data and were advocating that their countries revise their guidelines for fluid management of shock. They requested that WHO do the same.

## 2013 WHO guidelines in practice

WHO had begun revising its *Pocket Book of Hospital Care for Children* when the FEAST trial results were released. We were aware of this process and provided additional unpublished data to the guideline developers when requested. We assumed that our data would be taken into account in the revision. However, the 2013 edition continues to recommend a 20 ml/kg bolus of isotonic crystalloid as fast as possible to any child fulfilling the WHO definition of shock, with up to two more boluses (that is, a total of 60 ml/kg) if shock fails to correct. 1 18 This is much more aggressive treatment than in the FEAST trial, where most children received a single bolus of 20 ml/kg over one hour.^2^

For children with suspected malaria or anaemia with shock, the new WHO guidelines state that “fluid be administered cautiously, and/or blood transfusion should be given for severe anaemia,”^1^ leaving clinicians unclear about the rate and volume of fluids to give in these two conditions. The guidelines committee did not consider the speed of resuscitation, only the choice of fluid.^18^

We are concerned that, given results of FEAST and their consistency across subgroups, including in those meeting the strict WHO definition of shock, these recommendations might expose substantial numbers of children to harm.

How many children do these guidelines apply to in Africa? There are no reliable data on the number of child admissions to hospital with shock each year in sub-Saharan Africa. We have previously reported that about 10% of children admitted to hospital in the coast of Kenya present with shock,^19^ indicating that the number would likely run into millions. For every million hospital admissions with shock, around 20 000 (2%) would be expected to meet the WHO definition of shock.^2^ Our subgroup analysis of the FEAST results suggested bolus was associated with a relative risk of death of 240% in these children. Treatment with rapid fluid resuscitation may therefore result in hundreds or thousands of excess deaths.

Distinguishing between WHO defined shock and other milder forms of shock is challenging in practice. Accurate measurement of blood pressure in children requires training to use automated technologies that are expensive, require frequent maintenance, and are rarely available. Capillary refill is difficult to measure accurately and has inherent between and within observer variation.^20^ WHO does not give advice on how to manage children who do not meet its definition of shock, and it is likely that there will be slippage in the implementation of the guidelines, as there is in high income countries, with children who do not meet the strict definition being given rapid fluid resuscitation. This could expose even more children to the harmful effects of fluid boluses.

The failure of WHO to take account of the FEAST data is disappointing and puzzling, particularly given its commitment to systematic assessment of evidence. Indeed, the pocketbook’s guidance on managing severe malaria was amended in the light of a trial showing the benefit of artensuate that was published in 2010,^21^ shortly before FEAST. We call on WHO to urgently reassess the evidence for bolus fluid resuscitation and revise its guidelines in accordance with this assessment.

## Figures and Tables

**Figure:**
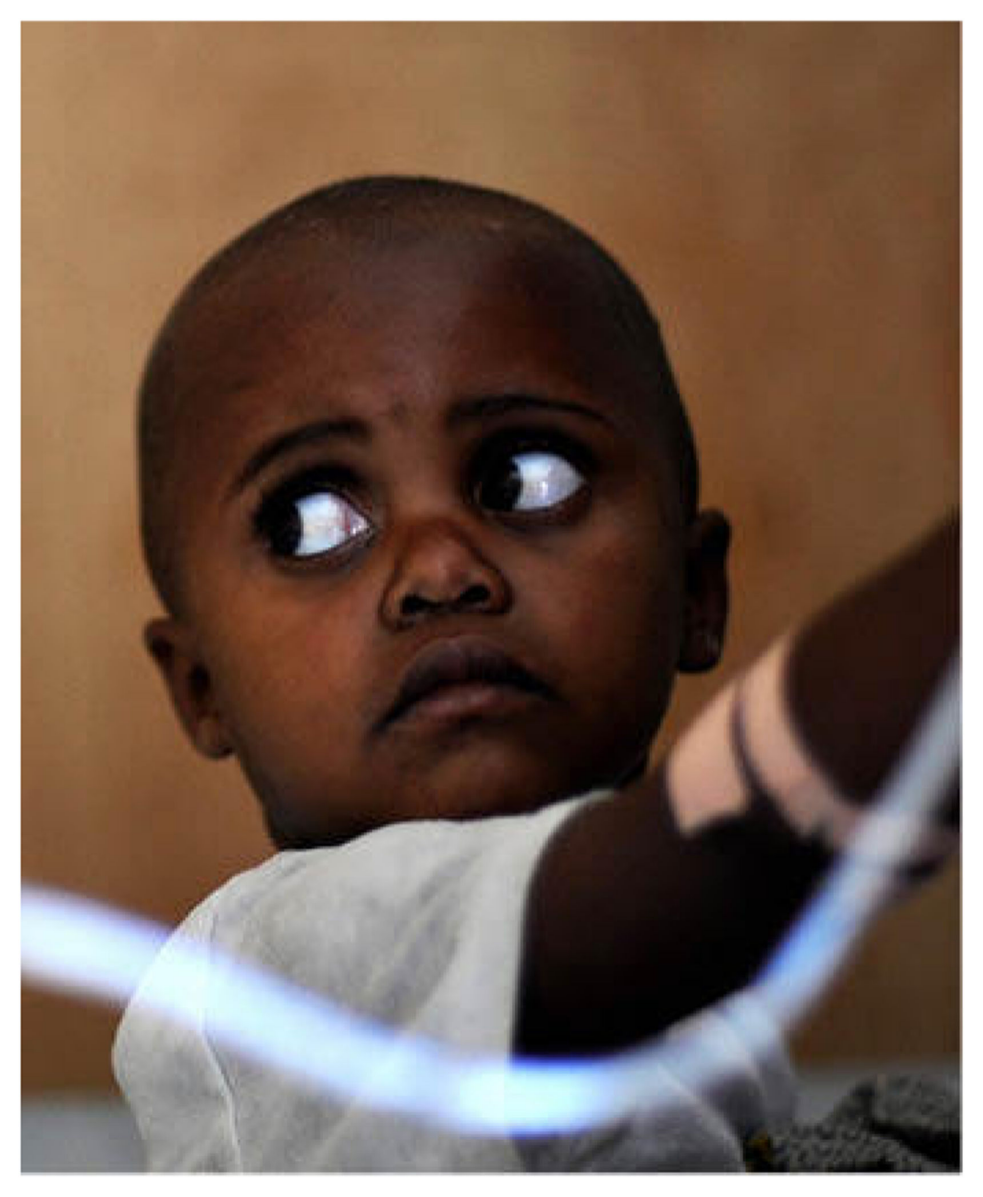
RPBERTO SCHMIDT/AFP/GETTY IMAGES

**Table 1:** Risk of death among participants in the Fluid Expansion as a Supportive Treatment (FEAST) trial with the application of different definitions of paediatric shock to admission data

Definition of shock	Mortality among FEAST participants (%)			Absolute risk difference (95% CI)	Estimated annual No of excess deaths in sub-Saharan Africa if boluses given*
	Overall (all arms)	Bolus (saline or albumin)	No bolus (control arm)		
**FEAST inclusion criteria**					
Total	297/3141 (10)	221/2097 (11)	76/1044 (7)	3.3% (1.2 to 5.3)	33 000
With malaria	144/1795 (8)	110/1202 (9)	34/593 (6)	3.4% (0.9 to 5.9)	14 500
Without malaria	146/1330 (11)	108/884 (12)	38/446 (9)	3.7% (0.3 to 7.1)	16 000
**WHO Emergency Triage Assessment and Treatment**					
Total	27/65 (42)	24/50 (48)	3/15 (20)	28% (3 to 53)	1 800
With malaria	14/41 (34)	12/32 (37)	2/9 (22)	15% (−16 to 47)	1 300
Without malaria	11/22 (50)	11/17 (65)	0/5 (0)	65% (42 to 87)	3 100
**American College of Critical Care Medicine cold shock (with two signs)**					
Total	189/1733 (11)	147/1196 (12.3)	42/537 (8)	4.5% (1.5 to 7.4)	14 300
With malaria	95/1087 (9)	76/753 (10)	19/334 (6)	4.4% (1.1 to 7.7);	8 900
Without malaria	92/637 (14)	70/435 (16)	22/202 (11)	5.2% (−0.3 to 10.7)	9 900
**Paediatric Advanced Life Support (2010) compensated shock**					
Total	218/1650 (13)	161/1113 (15)	57/537 (11)	3.9% (0.5 to 7.2)	26 300
With malaria	107/1009 (11)	80/684 (12)	27/325 (8)	3.4% (−0.4 to 7.2)	12 000
Without malaria	104/628 (17)	78/421 (19)	26/207 (13)	6.0% (0.1 to 11.8)	11 300

* Per 1 million paediatric admissions with shock, using relative increase of 1.45 from overall trial result. NB: There are 16 children with missing malaria results who are not included in the with/without malaria calculations. FEAST criteria: History of fever or axillary temperature >37.4°C or <36°C with impaired consciousness (prostration or coma) or respiratory distress. plus ≥1 of the following: capillary refill time >2 s, lower limb temperature gradient, weak pulse, tachycardia (heart rate >180 (<12 months), >160 (12 months-5 years), >140 (>5 years)). WHO Emergency Triage Assessment Treatment criteria: The presence of cold hands or feet with capillary refill time longer than 3 seconds and a weak, fast pulse. ACCM cold shock (with two signs): Axillary temperature >37.4°C or <36°C) plus ≥2 of: prostration/coma or Blantyre coma score <5, capillary refill time >2 s, weak pulse, increased temperature gradient. PALS (2010) compensated shock: Two of the following: tachycardia (see FEAST criteria for definition), increased temperature gradient, capillary refill time >2 s, weak pulse.
